# Skin Immune Landscape: Inside and Outside the Organism

**DOI:** 10.1155/2017/5095293

**Published:** 2017-10-18

**Authors:** Florence Abdallah, Lily Mijouin, Chantal Pichon

**Affiliations:** ^1^Centre de Biophysique Moléculaire, CNRS UPR4301, Orléans, France; ^2^Remedials Laboratoire, 91 rue du Faubourg Saint-Honoré, 75008 Paris, France; ^3^Collégium Sciences et Techniques, Université d'Orléans, Orléans, France

## Abstract

The skin is an essential organ to the human body protecting it from external aggressions and pathogens. Over the years, the skin was proven to have a crucial immunological role, not only being a passive protective barrier but a network of effector cells and molecular mediators that constitute a highly sophisticated compound known as the “skin immune system” (SIS). Studies of skin immune sentinels provided essential insights of a complex and dynamic immunity, which was achieved through interaction between the external and internal cutaneous compartments. In fact, the skin surface is cohabited by microorganisms recognized as skin microbiota that live in complete harmony with the immune sentinels and contribute to the epithelial barrier reinforcement. However, under stress, the symbiotic relationship changes into a dysbiotic one resulting in skin disorders. Hence, the skin microbiota may have either positive or negative influence on the immune system. This review aims at providing basic background information on the cutaneous immune system from major cellular and molecular players and the impact of its microbiota on the well-coordinated immune responses in host defense.

## 1. Introduction

Remarkable advances have been achieved over the past years to understand and characterize the immunobiology of the skin. As the largest organ of the integumentary system, the skin covers the internal organs of the body to maintain its temperature, to prevent water loss, and to provide a physical barrier against external insults. Far from being a simple mechanical barrier, the skin constitutes a network of effector cells and molecular mediators that constitute a highly sophisticated “skin immune system” (SIS) as described by Bos and Kapsenberg in 1986 [1]. The cutaneous homeostasis maintenance is dependent on the cross-talk between several immune sentinels present in the different compartments of the skin and the interplay between innate and adaptive immune responses. This SIS includes resident cellular players (keratinocytes, Langerhans cells, fibroblasts, mast cells, macrophages, endothelial cells, or recruited leucocytes) and a wide variety of soluble inflammatory mediators (antimicrobial peptides (AMPs), cytokines, and chemokines). This system allows the maintenance of cutaneous homeostasis and is also responsible for the activation and regulation of normal and pathological inflammatory reactions. More recently, the dynamic cutaneous ecosystem was shown to affect profoundly the immune response. Moreover, the skin forms a complex and dynamic ecosystem colonized by about 10^12^ microorganisms including bacteria, fungi, and viruses known as skin microbiota. These organisms play an important role in the protection against invading pathogens and in the development of inflammatory-mediated diseases. Taken together, the total skin environment favors the interaction between the immune cells and the host microbial community. It results in a highly defined and organized defense response that can be divided into three major steps: (1) interplay of the cutaneous ecosystem and pathogen invasion, (2) onset of the immune response, and (3) immunological memory.

## 2. Step 1: Interplay of the Cutaneous Ecosystem and Pathogen Invasion

The skin is the first and largest barrier of the human body. It covers the human organism and ensures a constant dialogue with the external environment full of exogenous factors, such as foreign pathogens, ultraviolet (UV) radiation, and allergenic and chemical irritants. Therefore, through evolution, a dynamic cutaneous ecosystem has been developed in order to protect the host from undesirable insults and aggressions. This ecosystem comprises (1) a sophisticated immune system and (2) a normal flora inhabited by many commensal microorganisms such as bacteria, fungi, and viruses that constitute a cutaneous microbiome known as skin microbiota. Both are interconnected and mutually regulated to form a biological and immunological barrier. In this paragraph, we will review briefly the features of the skin microbiota and the mechanism by which commensal microorganisms such as *Staphylococcus epidermidis* and *Staphylococcus aureus* and fungi such as *Candida albicans* and *Malassezia* spp. invade the skin and become pathogenic.

**Figure 1:**
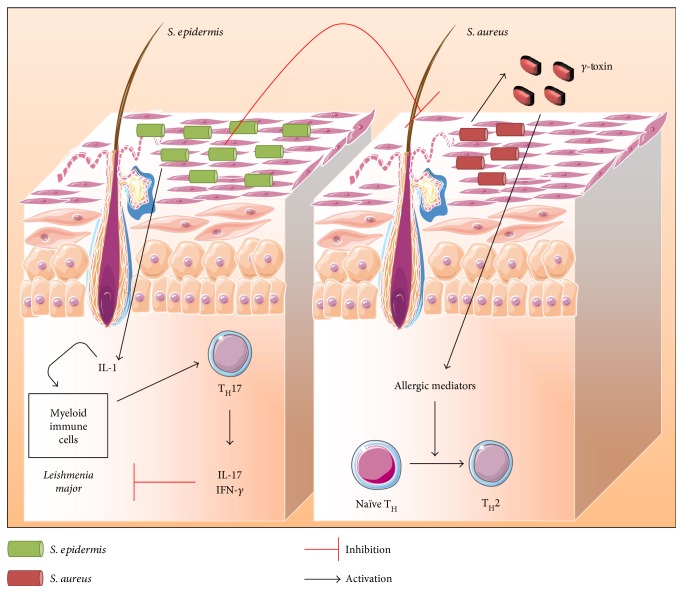
Illustration of skin microbiota interaction with the SIS. The alliance between skin immunity and local flora allows protection against external pathogens and maintenance of tolerance to resident microorganisms, simultaneously. In a healthy state, resident bacteria such as *S. epidermidis* are able to contain *S. aureus* pathogenicity. Moreover, *S. epidermidis* was shown to be required for the production of IL-17 and IFN-*γ* by T cells inhibiting *L. major* growth. However, altered resident microbial communities or local expansion of some members of skin microbiota with harmful potential alters this alliance. *S. aureus* is able to produce *δ* toxins that trigger local allergic responses in skin resulting in T_H_2 inflammatory immune response. Adapted from [157].

The skin microbiota extends from the skin surface to deeper layers in the dermis and dermal adipose tissue. Almost 25% of skin microorganisms grow in the dermis at the level of the sebum glands and through hair follicles [2]. These microorganisms are classified as resident and transient microorganisms [3, 4]. The resident microorganisms transmitted during birth from the mother or acquired from the contact with the daily life surroundings (animals, plants, persons, chemicals, and climates) are long lasting. On the other hand, the exposition to new settings (e.g., changes in the environment due to travelling) leads to the development of transient microorganisms that is eradicated once back to usual conditions. Therefore, each individual has a unique and specific signature of skin microbiota encountered during infancy and stabilized during adulthood [5, 6]. This skin microbiota lives in symbiosis with the SIS actors forming a strong biological shield against pathogens [4]. Hence, the resident flora has evolved tightly with the host gaining ability to train, to induce, and to modulate local immune reactions when appropriate [7]. For instance, the protection is acquired either directly through bacteriocin production, pathogen adhesion inhibition, and toxin degradation or indirectly through interaction and activation of the host SIS. In the last decades, studies have shown the importance of commensal microbes to promote immune development and to prevent infection without inducing detrimental inflammatory responses. The interconnection of the microbiota and the development of the SIS was pointed out [8]. Previous studies have demonstrated the importance of repopulating the skin with commensal microbes to restore an effective immune response against invading pathogens. For instance, *S. epidermidis* colonization in germ-free mice is sufficient to restore effective T cell immunity to parasites such as *Leishmania major* through modulation of IL-1, IL-17, and IFN-*γ* inflammatory response [9] (Figure 1). Moreover, lipoteichoic acids (LTA) present in the cell wall of *S. epidermidis* inhibit *Propionibacterium acnes*-induced inflammation via miR-143 induction [10]. *S. epidermidis* is also able to enhance host defense mechanisms by inhibiting the growth of group A *Streptococcus* and *S. aureus* [11, 12] (Figure 1).

*S. epidermidis* is also able to restrain *S. aureus* pathogenicity [13, 14] (Figure 1). In addition, *Malassezia* spp. is one of the most dominant cutaneous fungi that possess a protective role against bacteria and fungi in the skin via its antimicrobial activity. *Malassezia* spp. growth depends on processing of external lipids by enzyme production to yield short fatty acids such as azelaic acid. The latter is responsible for the antimicrobial activity in normal pH skin [15]. As mentioned earlier, the skin is a complex and dynamic environment. Its complexity derives from the intricate relationship existing between microbiota and immune responses. Commensal microbes colonize different areas of the skin surface that would be otherwise available for pathogens. So far, the cutaneous microbiota is an essential partner in protecting the skin from pathogen invasion [16]. Nevertheless, for many reasons, the protective symbiotic effect can turn into harmful and devastating opponents of the SIS leading to dysbiosis responsible for infection genesis and/or cutaneous disease development. There are several causes of symbiosis to dysbiosis shift. For instance, a modified immune tolerance and disrupted microbial homeostasis such as cutaneous lesions (open wound, catheter, burns, and insect bite), extensive scrubbing, hormonal deregulation, other environmental factors (antibiotics, cosmetics, and cold), or genetic predisposition/alteration may weaken the microbial barrier and modify the composition or the virulence of microbial communities, thereby facilitating the genesis of infection [17–19]. Indeed, in optimal conditions, the alliance between skin immunity and local flora allows simultaneously the protection against the external pathogens and tolerance maintenance towards resident microorganisms. Conversely, altered resident microbial communities or harmful local expansion of some members of skin microbiota can terminate this alliance. Clear evidences have been reported to prove the link between dysbiosis and skin diseases or infections, albeit the mechanisms are not yet fully understood [20]. Studies showed that dysbiosis is a major trigger of acute or chronic inflammatory disorders such as atopic dermatitis, acne, and rosacea [16, 21]. For example, *S. aureus* produces *δ* toxins triggering local allergic cutaneous responses which may also prevent wound healing and cause epithelial barrier deterioration [3, 22] (Figure 1). The correlation between skin immune disorders and microbiota will be developed in the last part of this review. Any shift among these populations can lead to aberrant skin immune responses. It is a vicious cycle: chronic and/or excessive immune responses can modify the composition of resident microbiota, allowing the attraction of new invasive microorganisms. These reactions can be further amplified by a positive feedback which leads eventually to a loss of skin homeostasis and to numerous pathologies [7]. In the following paragraph, examples of skin infections caused by skin colonizers are described.

*Staphylococci* are common bacterial colonizers of human skin [23], hence associated with high occurrence of skin infection breaking through the barriers. *S. epidermidis*, in particular, is the most frequent microorganism isolated from human epithelia and is an essential member of skin resident microflora [24]. In normal conditions, *S. epidermidis* has a sane adaptable relationship with its host. Nonetheless, its ability to form biofilms renders it highly resistant in case of infection. Biofilm-associated infections are extremely hard to clear, due to the difficulty to bypass the extracellular matrix [25]. This matrix acts as (i) a physical barrier restricting many antibiotics and chemical diffusion and (ii) a mechanical barrier restraining immune cell passage. Only a few cells, like neutrophils, are able to bypass this barrier, by using hydric channels, to penetrate the matrix and access bacteria [25]. In general, the host's immunity is not sufficient to clear off biofilm-associated infections thereby endorsing chronic infection. *S. aureus* is part of the human transient microflora but is considered “semipermanent”, since 30 to 50% of the human population are believed to be healthy carriers of *S. aureus* [26, 27]. Moreover, the skin of patients suffering from inflammatory chronic diseases initiating epithelial barrier disorders (e.g., atopic dermatitis) is often colonized by *S. aureus* [28]. As mentioned previously, a breach at the cutaneous level grants an easy and rapid access for microorganisms to deep tissues and the bloodstream and risks of the dissemination of the infection. Most common *S. aureus* infections include those in the skin and soft tissues, and the infection risk is enhanced by the use of medical implants, such as prosthetic joints and intravascular catheters [29]. In the late ‘60s, there was an emergence of methicillin-resistant *S. aureus* (MRSA) [30]. The virulence of this pathogen still poses a significant therapeutic challenge [31]. To date, the majority of research in the *Staphylococcus* field is dedicated to the understanding of *S. aureus* infection occurrence. The interference of *S. aureus* with the host's immune responses has been well described over years but remains quite dependent on the models [32]. The host's defense against *S. aureus* includes (i) the skin barrier and outcompetition with other strains, for example, *S. epidermidis*, as described previously; (ii) the innate immune responses, mostly driven by antimicrobial peptide (AMP), complement, neutrophil, and macrophage activation; and (iii) the adaptive immune response. *S. aureus* is an excellent model of bacteria being part of a semiresident flora, but able to switch as a pathogen as soon as it is left uncontrolled by other members of resident flora [33–36]. The coevolution of this particular microorganism with the host's SIS and its ability to get specific virulence genes easily and rapidly makes it a quite interesting target to understand how this system is dynamic.

More recently, there is increasing awareness of the importance of fungi and their interactions with the immune system influencing the immune homeostasis and inducing disease. When the chemical composition (pH, pathological sweat secretion) of host epidermis is disrupted, *Malassezia* spp. gains in pathogenicity and releases lipases, phospholipases, and an array of bioactive indoles. These molecules alter the function of the epithelial barrier resulting in immune deregulation and diseases [37, 38]. Another common cause of fungal infections worldwide is *Candida albicans* [39] despite being in most cases harmless commensal fungi. The dryness of the skin renders hard the growth of *C. albicans* and their cutaneous concentration remains low, yet a normal constituent of the resident skin microflora is about 70% of the population [40, 41]. This fungus is unable to cause severe disease when present at low rates, but inappropriate immune response or disruption in the normal floral occupancy can cause uncontrolled proliferation of this germ on the skin, thereby leading to cutaneous invasion and infection. *C. albicans* interacts with the host's defenses in three major ways: (i) innate response, (ii) adaptive response, and (iii) neuronal response [42–44].

## 3. Step 2: Onset of the Immune Response

### 3.1. Innate Immune Response

**Figure 2:**
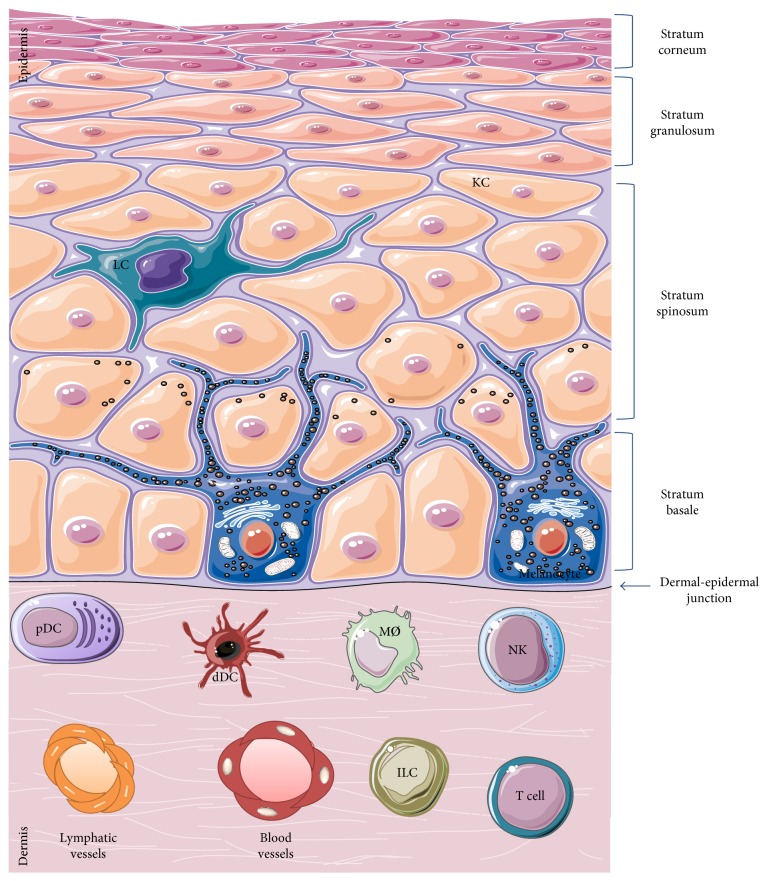
Skin anatomy and cellular constituents. The protection of the body from the external environment is provided by the multilayered structure as well as by the complex cellular composition of the skin. The epidermis is the outermost layer composed of different strata made of keratinocytes (KC) from the most exposed surface to the least differentiated deeper area: stratum corneum, stratum granulosum, stratum spinosum, and stratum basale. Immune cells that ensure immunosurveillance such as Langerhans cells (LC) and specialized cells that produce melanin such as melanocytes are found in the epidermis. The dermis is the intermediate layer composed of several specialised immune cells such as plasmocytoid dendritic cells (pDC), dermal dendritic cells (dDC), macrophages (MØ), natural killer cells (NK), innate lymphoid cells (ILC), and T cells responsible of the immune response. In addition, blood and lymphatic vessels are present throughout the dermis. The hypodermis (not represented) is the innermost layer constituted mainly of adipose tissue.

The innate immune system is designed to directly and rapidly respond to foreign pathogens by activating recognition systems and effector mechanisms (Figure 2). The major innate immune cells are macrophages, dendritic cells, and natural killer cells that express a wide variety of pattern recognition receptors (PRR) including two transmembrane proteins, Toll-like receptors (TLRs) and C-type lectin receptors. They also express two cytosolic proteins: retinoic acid-inducible gene-I-like receptors and NOD-like receptors (NLRs) [45, 46]. The most well-characterized PRR is the TLR family composed of 11 and 12 members in humans and in mice, respectively. TLRs detect a broad range of pathogen-associated molecular patterns (PAMPs) and conserved microbial sturctures, including lipopolysaccharide (LPS), peptidoglycan, flagellin, and nucleic acid ligands. TLR signaling is characterized by the activation of the critical transcription factor “nuclear factor kappa B (NF-*κ*B)” and mitogen-activated protein kinase (MAPK) pathways through adaptor proteins including MyD88, TIRAP/Mal, TRIF, and TRAM [47]. Consequently, genes involved in inflammatory responses (a panel of AMP, cytokines, and chemokines) such as IL-6, TNF-*α*, IL-8, and IL-12 are upregulated [45, 47]. The resulting inflammatory environment stimulates the neighboring cells to produce more inflammatory mediators and attracts innate immune cells to the stressed site. These recruited cells induce reactive oxygen species (ROS) and nitrogen oxide species (NOS) production [45, 47]. They also promote cell lysis and phagocytosis and boost cell autonomous defenses such as apoptosis to eliminate invaders [48, 49].

The ability of the innate immune cells to communicate with epithelial cells leading to an effective immune response is a key feature of the cutaneous immune system. It is of great importance to understand the cellular and structural composition of the skin that dictates the hierarchy of the skin immune response. Therefore, a quick brief overview of the constitution of the skin is essential before developing the steps of the cutaneous immune response.

**Figure 3:**
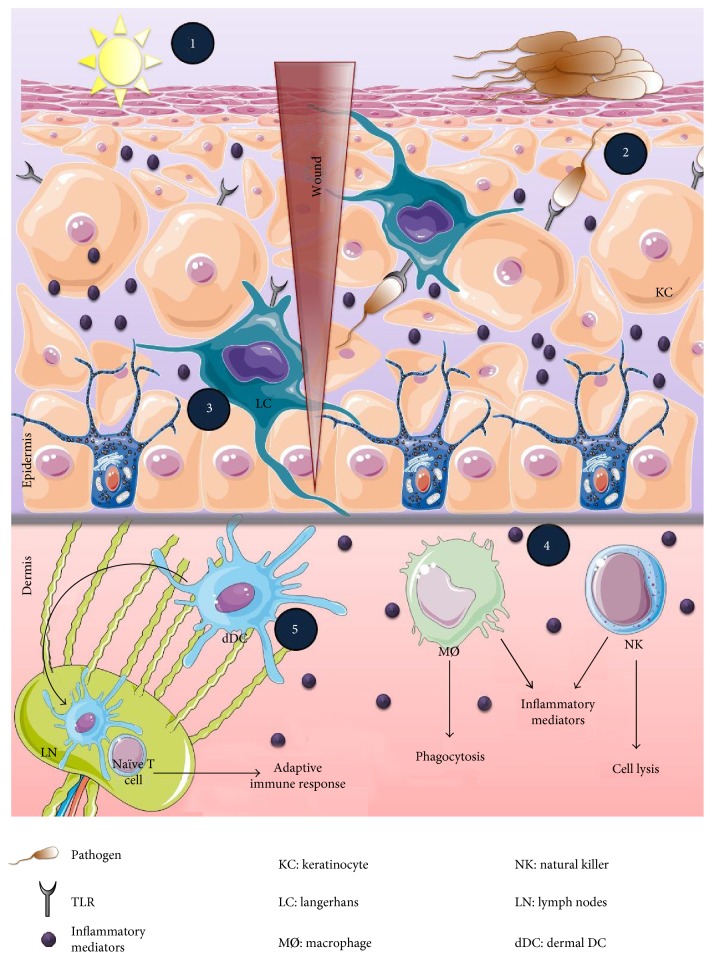
Initiation of a primary cutaneous immune response. The skin is a primary immunological barrier to the external environment. The uppermost layer “corneal layer” is composed of dead keratinocytes that provide a physical barrier. However, the pathogens can access directly to the interior of the host through skin wounds and by outcompeting the normal flora (1). TLR-bearing cells (KCs and LCs) recognize pathogens and establish a highly coordinated immune response: antimicrobial production to neutralize the pathogen (2), inflammatory mediator secretion to alert the immune cells (3), activation of innate immune cells such as natural killer cells (NK) to induce cell lysis and/or phagocytosis such as macrophages to engulf pathogens (4), and maturation of dermal DCs that migrate into draining lymph nodes to prime T cell responses (adaptive immunity) (5).

The skin is made up of various cell types, each characterized with specific functions according to their location. It has three layers: (i) the epidermis, the outermost layer containing predominantly keratinocytes and, to a lesser extent, melanocytes, CD8^+^ T cells, and Langerhans cells with a simple cell composition; (ii) the dermis, the intermediate layer with greater cell diversity—dendritic cells, macrophages, natural killer cells, CD4^+^ T cells, innate lymphoid cells, fibroblasts, and so forth—and with lymphatic and blood vessels which allow cell migration traffic [50]; and (iii) the hypodermis, the innermost layer, composed mainly of adipocytes, which ensures thermoregulation. The epidermis is separated from the dermis by the dermoepidermal junction and from the external environment by the stratum corneum (Figure 3). The latter represents a true barrier of protection. It is composed of cells made up mainly of proteins called corneocytes, whose intercellular space is highly constituted of lipids. The dynamic interaction between all these cells coordinates the immune response.

The first sensors of pathogen invasion are *keratinocytes (KCs)* which represent 95% of the epidermal cell type and ensure its structural integrity [51]. The corneal layer made of dead KCs constitutes the skin's mechanical barrier. KCs are the initiators of the immune response [52] and thus could be perceived as immune sentinels. KCs of the granular spinous and basal layer can sense nonspecific external stimuli such as UV rays and chemicals and detect a wide range of microbial ligands via TLRs expressed on their surface. So far, TLR1, 2, 3, 4, 5, 6, and 9 have been shown to be expressed in a constitutive or inducible manner in KCs [53–58]. As a response to stimulation, KCs produce a wide panel of cytokines (IL-1, TNF-*α*, IL-6, G-CSF, TGF-*β*, and IL-10), chemokines (CXCL-8, IP-10), growth factor (IL-6, GM-CSF, and TGF-*α*), and AMPs (*β*-defensins, cathelicidins, S100 family members, and sebum) resulting in either direct neutralisation of the pathogen or indirect activation of other immune sentinels to launch a specific immune response [59] (Table 1). The nature of the immune response depends on the stimulus. For example, UV rays and chemicals activate the inflammasome-dependent proinflammatory signaling pathway leading to IL-1*β* secretion [60, 61], whereas a dominant T_H_1 immune response accompanied with type 1 interferon (IFN) production is obtained upon PAMP-TLR pathway activation. This elicits the cell-mediated immunity against infection [62]. Furthermore, another immunological function of the KCs has been described in ingraft versus host disease. Nickoloff and Turka demonstrated that MHC class II-expressing KCs act as nonprofessional antigen-presenting cells that are able to activate and maintain T cell tolerance [63]. The costimulatory pathways (BB1 and B7-H1) initiated by KCs differ from those coming from professional cells (B7-1 and B7-2). Taking all described differences into consideration, T cells' interaction with KCs remains crucial in mounting the immune response to local antigens and also in maintaining self-tolerance. Note that KCs can also interfere with the adaptive immune response; they are playmakers in coordinating immune responses due to their ability to cross-talk with other epithelial and immune cells.

*Melanocytes* are epithelial cells recently described for their potential in modulating the immune response through inflammatory cytokine production. They are mostly located in the epidermal basal layer towards the dermoepidermal junction. They are oval, fusiform, and smaller than KCs. The expression of melanocyte-specific proteins such as tyrosinase (tyr), TYRP1, DCT, Pmel17/gp100, MART-1, and/or MITF allows differentiated melanocyte identification. The main features of these cells are melanin production and melanosome transfer from differentiated melanocyte to KCs. The melanin presence in the skin defines its pigmentation and is involved in the photoprotection against UV rays [64]. The contact between KCs and melanocytes is crucial, and the underlying molecular mechanisms are still a subject of investigations [65]. Besides melanogenesis, the role of melanocytes in the inflammatory response is minimally studied although they have been described to produce various inflammatory cytokines.

*Fibroblasts* are also implicated in the immune response via their interaction with KCs. They are the main cellular constituents of connective tissues. These cells are major contributors in extracellular matrix (ECM) protein synthesis, through collagen and fibronectin secretion, as well as remodeling, by the production of proteinases. Even though, they secrete a complex mixture of growth factors, cytokines, and chemokines, they still are not considered immune sentinels. Fibroblasts communicate with nearby cells through the paracrine and autocrine system. For instance, the fibroblast-keratinocyte interaction modulates the levels of MMP-2 and MMP-9 and their inhibitors resulting in a better healing quality at a late stage of the wound healing process [66]. Thus, the dialogue between fibroblasts and KCs via cytokines plays a fundamental role in generating skin immunity (Table 1).

In parallel, Langerhans cells *(LCs)* are the first immune cells that come in contact with skin-invading pathogens. LCs are in intimate association with KCs and represent 2 to 4% of the epidermal cell population with a half-life range between 53 and 78 days [67]. LCs have been described for the first time, 150 years ago by Langerhans [68]. They are specialized residents of skin dendritic cells (DCs) of hematopoietic origin derived from bone marrow [69]. LCs within the spinous layer demonstrate a dendritic morphology that extends through tight junctions to the stratum corneum where it can capture antigen without disturbing the epithelial barrier [70]. LCs express C-type lectins on their plasma membrane langerin (CD207) in mice [71] and CD1a in human [72] and Fc*γ* and Fc*ε* receptors [73]. These surface C-type lectins are PRRs that recognize mannosylated ligands found on the surface of a wide range of pathogens [46, 74] which leads to receptor-mediated endocytosis and trafficking to the Birbeck granule where they may participate in antigen processing [75]. Unlike conventional DCs, *in vitro* studies showed that LCs are weak stimulators of T cell responses and have phagocytic capabilities. However, during culture, they become mature by acquiring immunostimulatory activity with increased MHC-II molecule expression and decreased Birbeck granule number and phagocytic capacity [76]. They play a primary defense role by monitoring the presence of infection and damage within the epidermis. They have been found to be major contributors in inducing IgG to neutralize *S. aureus* during cutaneous infection [77]. Activated LCs capture antigens and migrate into draining lymph nodes where naïve T cells are activated. Yet, the definitive function of LC and its contribution in the adaptive immune response is not fully understood.

The optimal outcome of the innate immunity is to eliminate pathogens and prevent full-blown infections from happening. For this purpose, *macrophages* (MØ), phagocytic cells, play a key role in inflammation dampening and host defense activation. MØ control the immune response in three phases. During the first phase, MØ recognize the crystallized fragment (Fc) of IgG-covered microbes via Fc*γ*RI (CD64^+^) leading to pathogen destruction via antibody-dependent cell cytotoxicity (ADCC) and phagocytosis. Alternatively, microbes coated with the complement C3b are identified by MØ with the help of the complement receptor C3bR leading to their lysis or phagocytosis. In the second phase, MØ developed another strategy to destroy pathogens. It is based on proinflammatory mediator secretion including the production of ROS and nitric oxide (NO), as well as proinflammatory cytokine secretion such as TNF-*α*, IL-6, and IL-1*β* (Table 1). The duration of this proinflammatory phase depends on the balance between the capacity of the microorganism to survive and the capacity of MØ to remove them. Finally, the last phase is meant to suppress inflammation and to improve apoptotic body removal. It involves anti-inflammatory mechanisms triggered by TGF-*β* and lipid mediator production [78, 79].

When the innate immunity and signaling are insufficient to clear off a pathogen and to resolve pathogen invasion, the adaptive immune system kicks in. The quantity and the quality of an adaptive immune response depend on the strength of the innate immune response. Although innate and adaptive immune responses are distinct, they are highly interconnected. The coordination between innate and adaptive immunity is assured by dendritic cells (*DCs*), which are professional antigen-presenting cells known as immune system gatekeepers. They were discovered in 1973 by Steinman and Cohn [80]. In 2011, Steinman was awarded by a Nobel Prize for his work that demonstrated that DCs play a crucial role in the immune system by linking the innate and the adaptive immunity. DCs represent a complex heterogeneous network of subsets that differ in ontology and specific functions. The first step in DC generation occurs in the bone marrow where two precursors committed to either conventional myeloid DCs (mDCs) or nonconventional plasmacytoid DCs (pDCs) were derived. The last step of DC differentiation is also dependent on the DC subset. For instance, mDCs undergo differentiation in the periphery whereas pDCs complete their development in the bone marrow. The maturity of DCs is highly dependent on pathogens they encounter. Different features of the DC population can be observed according to local environmental cues. Regarding the status of the tissue, steady or inflammatory, several subsets of resident or recruited DCs with differential phagocytic activity and capacity to produce cytokine could be identified. One common function among the heterogeneity of these cells is their antigen processing, and presenting cells implicated in T cell tolerance [81–83] make them strategic cells able to participate in both innate and adaptive immunity. DCs recognize antigen via a diverse array of TLR1, 2, 4, 5, 6, 11, and 12. *Dermal DCs* are divided into several subsets. In the mouse dermis, two resident DCs were identified in normal skin: CD103^+^CD11b^−^ (CD103^+^ DCs) and CD103^−^CD11b^+^ (CD11b^+^ DCs) [84]. They share functional homology with human CD141^hi^CD14^+^ DCs and Cd1a^+^CD1c^+^ DCs, respectively. Since these DCs have the power to catch cutaneous antigens, mature, and migrate to draining local lymph nodes, they become migratory skin DCs [85, 86]. The migratory DCs act as antigen-presenting cells (APCs) and are able to interact with antigen-specific lymphocytes such as T cells subsequently activating the adaptive immune response. Plasmacytoid DCs (pDCs**)** are quite rare in human skin; they are acting during viral infection by the production of large amounts of IFN-*α* via TLR7 and 9 activation [87, 88].

### 3.2. Adaptive Immune Response

**Figure 4:**
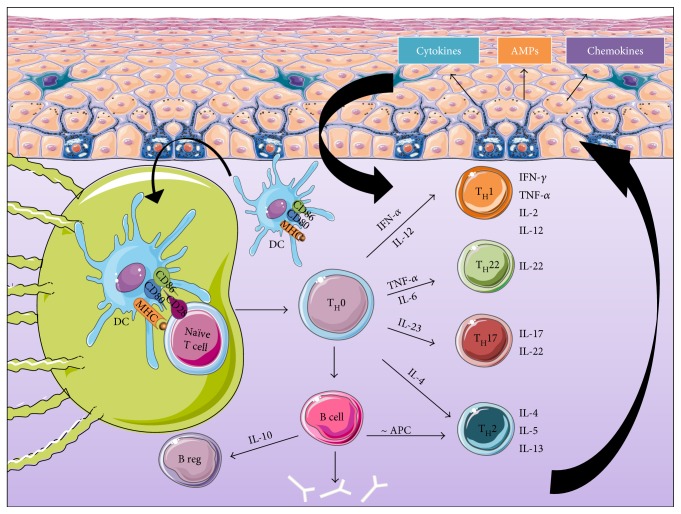
Major actors of the adaptive immune response. The adaptive immune system mounts a stronger, antigen-specific immune response when the innate immune response fails to eliminate pathogens. The first phase consists of the activation of antigen-presenting cells such as DCs allowing their migration into lymph nodes where they prime naïve T cells. Activated T cells migrate back to the site of infection where they induce cell-mediated and humoral immunity causing mediator release by the immune cells present at the site of infection. The resulting cytokinic environment stimulates epidermal cells mainly KCs to release further mediators that activate and maintain the dermal immune response. Hence, a positive feedback loop forms. DCs: dendritic cells; KCs: keratinocytes; TH: T helper; MHC: major histocompatibility class; IFN: interferon; CD: cluster of differentiation; IL: interleukin; TNF: tumor necrosis factor.

In contrast to innate immunity, the adaptive immune system provides a more delayed and specific response. A unique feature of the adaptive immunity is its ability to generate and to retain memory providing a more rapid response in the event of subsequent immunologic challenge. The adaptive immune response consists of humoral and cellular immune reactions carried by adaptive B and T cells, respectively. T cells are major contributors in safeguarding the cutaneous barrier. They are located next to papillary venules and beneath the dermoepidermal junction as well as adjacent to cutaneous appendages in the dermis. The activations of adaptive B and T cells through antigen-specific receptors demand antigen encounters either free antigen or bound antigen by APC to become effector cells. In the following paragraph, the mechanisms of B and T cell activation, maturation, and functions will be overviewed (Figure 4).

The activation of naïve T cells requires two signals provided by APC, mainly DCs. In fact, once inside the lymph nodes, DCs migrate to T cell areas, seeking out antigen-specific T cells by furnishing the necessary signals to induce their activation and differentiation into effector cells. The first signal is MHC molecules presented by DCs after microbial antigen capturing and processing. The second signal is the promotion of CD28 expressed on naïve T cells via costimulatory molecules B7-1 (CD80) and B7-2 (CD86) expressed on DCs (Table 2). The induction of CD80 and CD86 is a very crucial step, which is launched by microbial pathogen recognition [48]. Resident and recirculating T lymphocytes in the skin are a major subtype of leukocytes produced in the bone marrow and matured in the thymus. They are famous for their capacity to recognize a wide range of antigens due to their ability to rearrange the DNA encoding for their T cell receptor (TCR) [89]. Two types of T cells exist, *αβ*T cells and *γδ*T cells, which differ in the structure of TCR displayed on their membrane. The other variability is the cluster of differentiation expressed on the surface of T cells that result in two different subpopulations of T cells: CD8^+^ and CD4^+^ T cells. CD8^+^ T cells recognize antigenic peptides presented by MHC-I molecules on APC and are qualified as cytotoxic T lymphocytes (CTLs) responsible for cellular lysis through the secretion of enzymes (perforins and granzymes) that alter the cytoplasm of target cells (Table 2). CTLs use also other mechanisms to kill intracellular pathogens by triggering caspase activation leading to apoptosis. Also, CTLs produce TNF-*α* and IFN-*γ*, which have antitumor and antiviral microbial effects. CD4^+^ T cells are essential for both the T cell-mediated and antibody-mediated branches of the immune system. They recognize antigenic peptides presented by MHC-II molecules. T_H_ cells have been described to be differentiated into two subsets of conventional T cells T_H_1 and T_H_2 during inflammatory diseases. The differentiation depends on the cytokinic environment and the nature of the antigen (parasite, virus, bacteria, fungi, and extracellular or intracellular organisms). MØ and DCs release IFN-*α* and IL-12 that stimulate T_H_1 response resulting in IFN-*γ* and lymphotoxin secretion, recruiting phagocytic cells as MØ engulfing the intracellular pathogens [90] (Table 2). T_H_2 polarization is dependent on IL-4 liberation by naïve CD4^+^ T cells. T_H_2 response is important in the defense against large extracellular organisms such as helminths, utilizing cytokines such as IL-4, IL-5, and IL-13, promoting eosinophilia and mastocytosis (Table 2). Severe consequences occur when the balance between T_H_1 and T_H_2 is disturbed. T_H_1 can be associated with autoimmunity and chronic inflammatory disease such as psoriasis whereas T_H_2 can lead to allergic diseases such as atopic dermatitis [91–93]. More recently, two populations of CD4^+^ cells were identified: T_H_17 and T_H_22. APCs release IL-23 that results in T_H_17 differentiation. T_H_17 produces mainly IL-17 and IL-22 promoting immunity against various fungal and bacterial infections [94, 95] (Table 2). The differentiation of T_H_22 was promoted by TNF-*α* and IL-6 released from DCs. T_H_22 are a subset of circulating T cells with skin-homing potential that produce IL-22 but not IL-17 and IFN-*γ* [96, 97] (Table 2). Numerous skin disorders are caused by the deregulation of T_H_17 and T_H_22 immune responses leading to both psoriasis and atopic dermatitis [94, 98, 99].

The role of cutaneous B cells is poorly documented. In general, once naïve B cells encounter a circulating antigen in the periphery, they complete their maturation process. Mature activated B cells release antibodies into blood and tissue fluid in order to target antigens. When immunoglobulins cover the antigen, a series of different reactions can occur including complement activation and pathogen opsonisation in order to neutralize and evacuate pathogens. Moreover, activated B cells can serve as APC to prime T helper cells into T_H_2 mediating a humoral immunity. In 2002, Shlomchik and colleagues have demonstrated that B cells producing IL-10 have suppressive capacity and thus can be qualified as B regulatory cells (Breg) [100, 101] (Table 2). IL-10 is a key cytokine, and besides its suppressive function and anti-inflammatory virtue, it acts as a growth factor promoting B cell maturation into antibody-producing plasma cells [102, 103]. B cells have been found in skin dermis during chronic inflammations caused by cutaneous leishmaniasis, diffuse cutaneous sclerosis, and atopic dermatitis [104–106]. They play a role in cutaneous inflammation via interactions with both innate immune cells and T cells. More recently, B cells are found to inhibit Treg and T_H_17 responses via IL-10 production [107]. These cells were found to improve cutaneous inflammatory responses in murine models of skin inflammation [108]. They were also detected in the lymphocyte population of human skin and described as innate-like B cells that migrate from central reservoirs into the skin [109].

The diverse immune responses are cross-connected. A bridge between innate and adaptive immunity is required for a better infection resolution and an enhanced immunosurveillance. In fact, the inflammatory mediators released from the adaptive immune response stimulate epidermal cells mainly the KCs. They react in turn by secreting further mediators that can stimulate the dermal adaptive immune cells. A positive feedback loop is then formed to amplify the immune response. This coordination between the cells in the different compartments of the skin and those in the lymphatic and blood systems leads to neutralization of the pathogen. After being challenged, the SIS keeps a memory of the antigen nature, in case of a second exposition, to be more reactive and directly effective.

## 4. Step 3: Immunological Memory

The T cell-mediated immunity is the central element of the adaptive immune system as developed earlier in this review. Recall that adaptive immunity consists of three essential phases: T activation, effector function, and persistence “memory.” This paragraph focuses on skin resident T cells that belong to the memory T cell subset. The T cells arrive to the skin after pathogen challenge and are maintained as memory populations. They are sustained by growth factors supplied by KCs and other tissue resident cells [110]. In fact, skin resident T cells in healthy skin accounts for 2 × 10^10^ cells that correspond to nearly twice the number of T cells in the whole circulation [111]. This huge amount of skin resident T cells is necessary to afford immunosurveillance of the cutaneous barrier exposed continuously to external environment with a high risk of pathogen invasion. In other words, these memory T cells provide long-lasting and rapid responses to pathogen re-encounter. Among these memory T cells, a combination of resident and recirculating memory T cells exist [112]. Recently, Watanabe and colleagues developed a skin xenograft model (nude NGS mice were grafted with human neonatal foreskin) that allowed them to identify four distinct populations of memory T cells: two resident subsets—effector memory (T_EM_) and resident memory (T_RM_), and two recirculating subsets—migratory memory (T_MM_) and central memory (T_CM_) [112–115]. These subsets can be distinguished by their localization and functional activities. T_EM_ are the first actors during an immune response. They express high levels of CD44 and lack homing addressins (L-selectin and CCR7) [114, 116, 117]; thus, they are not circulating T cells and can be found in nonlymphoid tissues. They exert immediate effector function and secrete cytokines mainly interferon-*γ* and T_H_1, T_H_17, and T_H_22 proinflammatory cytokines [115]. However, these T_EM_ disappear once the infection is resolved leaving the place for T_CM_. By contrast to T_EM_ [118], T_CM_ express high levels of homing addressins (L-selectin, CCR7, cutaneous lymphocytes antigen “CLA,” and CCR4) [111, 119, 120], which allow their migration in both directions either in lymph nodes (LNs) or in skin. They can also produce IL-2 and T_H_2 cytokine (IL-4 and IL-13) [115]. Interestingly, upon rechallenge, persisting T_CM_ are activated in the LN where they extensively proliferate and convert into T_EM_ phenotype to assure an effective appropriate local immune response [114, 121]. Therefore, T_CM_ play a key role in maintaining long-lasting immunologic memory. Recent discoveries described a new powerful subset of resident memory T cells (T_RM_) that remain in tissues after infection ready to act in case of antigen re-encounter [122]. They have more potent effector function than circulating T cells and have limited proliferation properties. T_RM_ phenotype is quite similar to that of T_EM_. The emerging studies were able to highlight the importance and the efficacy of T_RM_ in providing an immediate and highly protective rapid local immunity_,_ though the molecular mechanism by which the T_RM_ are regulated is not fully understood. There are two subsets of T_RM_ including CD103^+^ cells which are enriched in the epidermis with increased cytokine production (IFN-*γ*, TNF-*α*, and IL-22) and CD103^−^ cells present in the dermis with a lower effector function [115]. Recently, Watanabe et al. defined a new subset of recirculating T cells T_MM_. They are CLA^+^, CCR7^+^, and L-selectin^−^, recirculating between the skin and LNs. Nonetheless, since they lack L-selectin, these cells are suspected to reside in the skin after infection resolution. They are considered an intermediate in cytokine production between T_CM_ and T_EM_ [115]. Further studies are needed for a better comprehension of these T_MM_.

## 5. Association of Skin Disorders with Cutaneous Microbiota Disturbance

In dermatology, antimicrobial agents are used to clinically improve several skin diseases. Therefore, scientists investigate the microbial contribution and association with different skin disorders. However, a direct causative relationship between a microbe and a disease remains partially identified. In fact, the four criteria of Koch's postulates are hard to satisfy in some cases. For instance, isolated microorganisms from sick skins are often considered commensal in steady-state condition. Hence, they fail to cause disease when introduced into a healthy organism. In the following part of this review, we illustrate the different ways in which skin disorders are due to skin immune response deregulation associated with microbiota dysbiosis and vice versa.

### 5.1. Skin Immune Disorder Correlation to Microbiota

Atopic dermatitis (AD) is a chronic T_H_2-type inflammatory skin disease associated with cutaneous hyperreactivity to environmental triggers [123]. It affects at least 15% of children and 3% of adults [124]. Patients suffer from relapsing eczematous lesions with severe pruritus. These lesions are characterized by inflammatory DC, MØ, and eosinophil infiltrations [125, 126]. AD is frequently connected with barrier dysfunction and transepidermal water loss associated with filaggrin gene (FLG) mutation that enhances susceptibility to microbial colonization such as *S. aureus* infections [127–129]. *S. aureus* itself is capable of penetrating the epidermis in case of increased cathelicidin expression and increased expression of IL-4, IL-13, IL-22, and other cytokines [127]. Flares of the disease are associated with an expansion of *S. aureus* on lesional skin and a substantial loss of biodiversity in skin microbiome [130]. However, the resolution of AD lesions is preceded by a restoration of microbial diversity demonstrating the implication of the cutaneous microbiota and AD development [130]. *S. aureus* release high levels of antimicrobial agents weakening other resident microorganisms and replacing them. *S. aureus* found in atopic skin produce toxins that contribute to inflammation and skin barrier dysfunction via host inflammasome activation. To cross the epithelial barrier, *S. aureus* promote peptidoglycan acetylation, superoxide dismutase, and catalase production to avoid phagocyte-mediated killing [35]. *S. aureus* escape the immune system by a plethora of secreted and surface-associated immune evasive molecules. *S. aureus* redirects host defense by fibrin formation or by disruption of adaptive responses, therefore preventing the establishment of protective immune responses [131–133]. Moreover, *S. aureus* adhesion to KCs stimulates their endogenous protease activity resulting in skin barrier integrity disruption [34]. KCs sense *S. aureus* via NOD-2 signaling activation and initiation of an IL-17 response, concomitant with AMP secretion [34, 134]. The bacterial replication within the skin will then induce the release of exoproducts (peptidoglycan, lipoproteins), which will promote inflammation and thus cytokine production [33] and dermal macrophage activation [135]. Indeed, macrophages will perceive these exoproducts via TLR and will activate neutrophil extravasation and migration to the site of infection [36]. Neutrophil adhesion and phagocytosis are then activated in IL-17-dependent T cell signaling [136]. The overall induced immune response aims at eliminating the bacteria. Until today, studies considering the different compartments/tissue layers populated by skin microbiome in AD have not been investigated in detail. These studies are crucial to develop new potential therapeutic targets.

### 5.2. Microbiota Correlation to Skin Immune Disorders

Acne vulgaris known as acne is one of the most common skin diseases that usually occur in puberty. Multiple factors can be at the origins of acne development in the sebaceous unit. The latter is colonized by *Propionibacterium* spp., a lipophilic bacterium that hydrolyses triglycerides present in sebum into free fatty acids resulting in skin acidification and emollition [5, 137, 138]. The correlation between *Propionibacterium acnes* and acne vulgaris has been well established since 1975 [139]. During puberty, increased sebum secretion induces proliferation of specific *P. acne*s subtypes, *S. epidermidis*, and *Corynebacterium* [140–144]. However, *P. acnes* relative abundance does not differ between individuals with acne and healthy ones [144, 145]. These findings raise the question how *P. acnes*, a commensal bacterium, functions as a pathogenic factor in acne. Metagenomic analysis demonstrated that certain strains were highly associated with acne and other strains were enriched in healthy skin [145, 146]. Thus, the pathogenicity and virulence of *P. acnes* are strain specific. Acne pathogenesis initiates and propagates due to abnormal keratinization resulting in pilosebaceous inflammation [147]. Other causes are attributed to a complex interplay of increased sebum productions, changes in the endocrine system, and local inflammatory cytokine secretion due to the activation of the innate immunity by *P. acnes* [147–150]. *P. acnes* mainly trigger the inflammatory process via TLR2 and TLR4 activation resulting in IL-1 secretion leading to KC hyperproliferation and further production of IL-1 [151]. As a matter of fact, IL-1 plays a key role in acne formation. It maintains an inflammatory milieu that boosts cellular proliferation and stimulates different cells such as neutrophils, endothelial cells, and follicular cells to generate further inflammatory mediators such as AMPs (*β*-defensin family, cathelicidin, and granulysin), cytokines (IL-1, IL-6, IL-10, IL-12, and TNF-*α*), chemokine (CXCL-8), matrix metalloproteinase (MMP-9), and NF-*κ*B [150–155]. In clinic, antibodies against *P. acnes* secretory factors were able to decrease acne inflammation demonstrating the essential role of *P. acnes* in acne-dependent inflammation. One of the novel approaches to treat acne is to supplement skin microbiota with healthful *P. acnes* strains or *S. epidermidis* known to inhibit pathogenic *P. acnes* growth as probiotic application (refer to Interplay of the Cutaneous Ecosystem and Pathogen Invasion) [156].

## 6. Conclusions and Perspectives

This review aims to give basic concepts of the skin immune system, the mechanisms underlying the immune response activation upon pathogen invasion, and the influence of skin microbiota on health and on disease. It is important to visualize the skin as a complex network of immune (innate and adaptive immunity) and epithelial cells that are in constant communication with the external environment and in effective activation of the internal environment (immune response) in order to maintain skin homeostasis. Although considerable attention was directed at the characterization of the interaction of the skin microbiome, there are much more factors that influence both the skin microbiota and the SIS. For instance, the age, sex, ethnicity, endocrine system, neurological system, and genetic predisposition are all contributors in both skin ecosystem and immune host defense. Despite the tremendous efforts made in this field, we are far from a full understanding of the immune regulation of the skin in health and in disease. More studies are needed to improve our understanding of the peaceful and mutual beneficial exchange between the host SIS and microorganism colonization. We are also far from understanding the global view and the cross-talk between the different axes of the SIS in a steady state and in a disorder state. Moreover, the translation of findings obtained from genetic mouse models into human skin poses a great challenge since there are fundamental differences between the mouse and human cutaneous composition and immune responses. Finally, the ultimate objective of studying the skin and the associated microflora is to find new efficient therapeutic invention against skin diseases, which constitute a large health and economic issues in the society.

## Figures and Tables

**Table 1:** Major constituents of the innate immunity.

Compartments	Cells	Inflammatory mediators
Epidermis	KC	AMPs, IL-1*β*, IL-8, IL-10
	LC	IL-1, TNF-*α*, IL-10, IL-15

Dermis	Fibroblasts	IL-6, TNF-*α*, IL-8, IL-1, MMP-9, MMP-2
	MØ	ROS, NO, L-1, IL-6, IL-8, IL-12, IL-10, TNF-*α*, TGF-*β*
	NKs	IL-4, IL-10

KC: keratinocyte; LC: Langerhans cell; MØ: macrophage; NK: natural killer; AMPs: antimicrobial peptides; IL: interleukin, TGF: tumor growth factor; TNF: tumor necrosis factor; MMP: matrix metalloproteinases; ROS: reactive oxygen species; NO: nitric oxide.

**Table 2:** Major constituents of the adaptive immunity.

Compartments	Cells	Inflammatory mediators

Dermis	DCs	MHC, CD80/CD86, IFN-*β*
	CTL	Enzymes, caspases
	T_H_1	IFN-*γ*, IL-2, TNF-*α*, IL-12
	T_H_2	IL-4, IL-5, IL-13 IL-4, IL-6, IL-15
	T_H_17	IL-17, IL-22
	T_H_ 22	IL-22
	B cells	IgA, IgE, IgG, IgD, IgM

DCs: dendritic cells; CTL: cytotoxic T lymphocytes; TH: T helper; MHC: major histocompatibility class; IFN: interferon; CD: cluster of differentiation; IL: interleukin; TNF: tumor necrosis factor; Ig: immunoglobulin.
